# Timing of Maternal Exposure and Foetal Sex Determine the Effects of Low‐level Chemical Mixture Exposure on the Foetal Neuroendocrine System in Sheep

**DOI:** 10.1111/jne.12444

**Published:** 2016-12-14

**Authors:** M. Bellingham, P. A. Fowler, E. S. MacDonald, B. Mandon‐Pepin, C. Cotinot, S. Rhind, R. M. Sharpe, N. P. Evans

**Affiliations:** ^1^ Institute of Biodiversity Animal Health and Comparative Medicine University of Glasgow Glasgow UK; ^2^ Division of Applied Medicine Centre for Reproductive Endocrinology and Medicine Institute of Medical Sciences University of Aberdeen Aberdeen UK; ^3^ UMR BDR Universite Paris Saclay Paris France; ^4^ James Hutton Institute Aberdeen UK; ^5^ MRC Centre for Reproductive Health University of Edinburgh Edinburgh UK

**Keywords:** GnRH, kisspeptin, oestrogen receptor, hypothalamus, endocrine disruptors, foetal

## Abstract

We have shown that continuous maternal exposure to the complex mixture of environmental chemicals (ECs) found in human biosolids (sewage sludge), disrupts mRNA expression of genes crucial for development and long‐term regulation of hypothalamic‐pituitary gonadal (HPG) function in sheep. The present study investigated whether exposure to ECs only during preconceptional period or only during pregnancy perturbed key regulatory genes within the hypothalamus and pituitary gland and whether these effects were different from chronic (life‐long) exposure to biosolid ECs. The findings demonstrate that the timing and duration of maternal EC exposure influences the subsequent effects on the foetal neuroendocrine system in a sex‐specific manner. Maternal exposure prior to conception, or during pregnancy only, altered the expression of key foetal neuroendocrine regulatory systems such as gonadotrophin‐releasing hormone and kisspeptin to a greater extent than when maternal exposure was ‘life‐long’. Furthermore, hypothalamic gene expression was affected to a greater extent in males than in females and, following EC exposure, male foetuses expressed more ‘female‐like’ mRNA levels for some key neuroendocrine genes. This is the first study to show that ‘real‐life’ maternal exposure to low levels of a complex cocktail of chemicals prior to conception can subsequently affect the developing foetal neuroendocrine system. These findings demonstrate that the developing neuroendocrine system is sensitive to EC mixtures in a sex‐dimorphic manner likely to predispose to reproductive dysfunction in later life.

It is evident from human and animal studies that many factors, including maternal nutrition and stress during pregnancy, can alter normal foetal development and programme risk of disease in later life 1, 2. The Developmental Origins of Health and Disease (DOHaD) paradigm has been one of the most rapidly expanding areas of biomedical research during the last decade 3. Maternal pregnancy exposure to exogenous chemicals/drugs either voluntarily [e.g. cigarette smoking and alcohol consumption 4, 5, 6, 7] or unintentionally [e.g. ubiquitous environmental chemicals (ECs)] is associated with altered foetal development leading to reproductive dysfunction in both males and females 8, 9, 10. Indeed, EC exposure is a candidate contributory factor to recently observed changes in human reproductive health, including an increased incidence of cryptorchidism and hypospadias 11, 12, 13 and reduced semen quality 14, 15, 16 in males. In females, the same link has been made to precocious puberty 17, 18, early menopause 19 and breast cancer 20, 21. Many ECs are classified as endocrine disrupting chemicals (EDCs), because they can enter the body and disrupt normal endogenous hormone release/action via a range of mechanisms 22. Some ECs can be stored in fat but are mobilised during periods of increased metabolism, such as during pregnancy. However, the potential impact of maternal chemical exposure prior to conception on subsequent foetal development has not been studied extensively.

When considering the factors that might influence the effects of ECs on foetal development, it is important to remember that humans are rarely exposed to high levels of individual chemicals but rather to mixtures of different chemicals. Such ECs are at low individual concentrations and potentially can have synergistic or additive effects 23 at the same time as also varying in concentration across gestation. Therefore, the physiological responses associated with exposure can differ according to the sensitivity of the foetus at the time of exposure (i.e. which critical developmental windows are affected) 24. Exposure to various exogenous or/and endogenous changes during specific windows of developmental programming may affect the long‐term health of the offspring with a disparity between males and females in the timing of onset and severity of disease outcomes, often with a long latency 25, 26.

To address the importance of pregnancy exposure relative to life‐long exposure to environmentally‐relevant, low levels of chemical mixtures, the present study builds on previously published work that used an ovine model of EC exposure, via grazing on pasture treated with human biosolids (sewage sludge). Biosolids, which are a by‐product of waste water treatment, contain a complex mixture of chemicals and pollutants with known endocrine disrupting capabilities 27. This complex mixture represents chemicals from anthropogenic sources and is thus reflective of everyday human chemical exposure from multiple sources. We have previously shown that life‐long maternal exposure to biosolids is associated with behavioural changes 28 and reduced bone density 29 in adult offspring, altered foetal testis and ovary development 24, 30, 31 and altered mRNA expression of regulatory systems within the foetal reproductive neuroendocrine axis 32, 33, whereas more recent studies have reported that the timing of maternal exposure has significant effects on foetal ovarian development 24, 34.

Reproductive success depends upon activity within the hypothalamic gonadotrophin‐releasing hormone (GnRH) neurosecretory system, which is dynamically regulated and highly sensitive to the organisational and activational effects of endogenous steroids 35, 36. The GnRH regulatory centres are therefore significant targets through which ECs may act to perturb reproductive function 37, 38. We have previously shown that life‐long maternal exposure to biosolids treated pastures results in altered expression of hypothalamic mRNA for GnRH, as well as afferent regulators of GnRH including galanin. More importantly, kisspeptin, the product of the *KISS1* gene and proposed gatekeeper of puberty, which plays a critical role in the steroidogenic regulation of GnRH 39, is also affected 32. The aim of the present study was to determine how the timing of maternal EC exposure, relative to pregnancy, impacts upon the expression of GnRH, oestrogen receptor (ER)α, KISS1 and KISS1 receptor (KISS1R) within the foetal reproductive neuroendocrine system.

## Materials and methods

### Ethics statement

All animals used in the present study were treated humanely with due consideration to the alleviation of pain, suffering, distress or lasting harm according to the James Hutton Institute's Local Ethical Committee and fully licensed by the United Kingdom's Animals (Scientific Procedures) Act 1986 under Project License authority (60/3356). All *in‐vivo* components of the study and euthanasia of animals were conducted at the James Hutton Institute under this legal framework operating at the highest ethical standards.

### Experimental animals, management and monitoring

The experimental design has been described previously 34, 40. Briefly, four experimental groups of ewes were set up in parallel: two groups of ewes (n = 12 per group) were exposed to either the biosolids treated (TT) or control (CC) pastures throughout their lives up to the time of mating and thereafter until the time of slaughter at 110 days gestation. In a cross‐over design, an additional group of ewes that had been raised on control pastures were transferred to the biosolids‐treated pastures 4 days prior to mating and maintained on these treated pastures until slaughter at 110 days of gestation (CT, n = 11) and a group of ewes that had been maintained on biosolids‐treated pastures throughout their lives were, 14 days prior to introduction of the rams (washout period to prevent faecal EC contamination of control pasture), transferred and subsequently maintained on control pastures (TC, n = 10) (See Supporting information, Fig. S1) as described previously 34, 40.

### Tissue collection

Prior to slaughter at 110 days of gestation, ewe body weight and condition was determined and a terminal blood sample taken. Ewes were euthanised by barbiturate overdose and foetuses were then removed, weighed and blood samples collected. Only one foetus per ewe was used for the present study to control for maternal or sibling influences. Maternal and foetal blood samples were centrifuged immediately and plasma was stored at −20 °C for hormone measurements. Hypothalami and pituitary glands were collected from foetal animals from each of the four maternal exposure groups, halved, frozen on dry ice and then stored at −80 °C until mRNA extraction and analysis. When still frozen, foetal hypothalamic blocks were cut into coronal slices (approximately 2 mm) as described previously 36. The most rostral slice was cut approximately 1 mm in front of the optic chiasm and encompassed the preoptic area (POA). A slice was also harvested approximately 1 mm dorsal to the mediobasal hypothalamus/median eminence, which encompassed the arcuate nucleus (ARC). From each of these tissue slices, approximately 20–30 mg of tissue was harvested, using a tissue punch, for RNA extraction from an area close to the ventricle that would encompass each of these two nuclei. Approximately 20–30 mg of tissue was also harvested from the mid sagittal face of the foetal pituitary gland for RNA extraction 33.

### RNA extraction

Total RNA was extracted from hypothalamic and pituitary gland tissue using TRIzol^®^ (Invitrogen, Carlsbad, CA, USA) in accordance with the manufacturer's instructions, and mRNA (200–300 ng) was reverse transcribed using Moloney‐murine leukaemia virus reverse transcriptase (Invitrogen), random hexamers (Promega, Madison, WI, USA) and Rnasin (Promega) as described previously 41. Purity and quantity of mRNA and cDNA were assessed using an ND‐1000 spectrophotometer (NanoDrop, Wilmington, DE, USA).

### Hormone measurements

Maternal and foetal plasma concentrations of oestradiol, testosterone, progesterone follicle‐stimulating hormone (FSH), luteinising hormone (LH) and inhibin A were estimated, in duplicate, as described previously 34 using protocols validated in sheep. For oestradiol, mean intra‐ and inter‐assay coefficients of variation (CV) were 8.5% and 6.15%, respectively, and the assay sensitivity averaged 0.27 pg/ml. The mean intra‐assay CV was 7.5% and assay sensitivities were 0.1 and 0.2 ng/ml for FSH and LH, respectively, and the assay sensitivity averaged 0.19 ng/tube. For plasma testosterone, the mean intra‐ and inter‐assay CV was 9.4% and 9.6%, respectively, over three assays and the assay sensitivity averaged 0.015 ng/ml. For progesterone, the mean intra‐ and inter‐assay CV was 1.9% and 3.2%, respectively, and the assay sensitivity was 0.67 nm.

### Quantitative PCR (qPCR)

mRNA expression in the hypothalamus and pituitary gland was quantified using SYBR green real‐time qPCR, in a 96‐well plate format using an MX3000 cycler (Stratagene, La Jolla, CA, USA). Reactions contained 5 μl of 2 × SYBRII mastermix (Stratagene), primer (100 nm) and template in a total volume of 10 μl. At the end of the amplification phase, a melting curve analysis was carried out on the products formed. mRNA expression of genes of interest was quantified using the comparative CT (cycle threshold) method 42 and gene expression was calculated relative to the reference gene (*β‐actin*). Primers for all genes were designed using primer express, version 2.0 (Applied Biosystems, Foster City, CA, USA) to span intron/exon boundaries and to have an annealing temperature of 65 °C.

### Statistical analysis

All data are presented as the mean ± SEM. Expression data were analysed using a generalised linear model where, within each hypothalamic region, the dependent variables were sex and treatment. Graphics were produced using r studio, version 2.15.0 (R Development Core Team, 2013) with the additional packages sciplot and pmcmr 43, 44. All of the explanatory variables were analysed for covariance and variance inflation and none were found. Foetus number per ewe was included as an explanatory variable and had no significant effect on the results. P < 0.05 was considered statistically significant.

## Results

### Maternal morphology and endocrinology

Data on maternal and female foetal morphology and endocrinology at day 110 of gestation have been reported previously as part of a related study 34 and are provided in the Supporting information (Fig. S2). Of relevance to the present study, maternal body condition scores were indicative of a normal nutritional state for the stage of gestation in all groups and there were no differences in number or in the sex ratio of foetuses produced between the four different exposure groups.

### Foetal morphology and endocrinology

As reported previously for females 34 and summarised in Table 1 (to allow comparison of sex differences), there was no significant effect of either treatment or sex on foetal gross morphology.

**Table 1:** Comparison of effects of of biosolids exposure on day 110 male (M) and female (F) fetuses: morphological and endocrine characteristics (female characteristics have been previously published 34. Values are mean±SEM

Treatment groups	Constant exposure profile		Cross‐over exposure profile	
Sex	CC(n = 12)	TT(n = 12)	CT(n = 11)	TC(n = 10)
Morphology				
Body weight (g)				
Female	1845 ± 50	1829 ± 84	1922 ± 84	1827 ± 73
Male	1979 ± 119	1966 ± 54	1801 ± 107	1911 ± 64
Endocrinology				
LH (ng/ml)				
Female	1.65 ± 0.33	1.79 ± 0.37	1.73 ± 0.75	2.49 ± 0.92
Male	2.5 ± 0.5^a^	1.9 ± 0.3	*2.8 ± 0.6* ^b^	*3.4 ± 0.8* ^b^
FSH (ng/ml)				
Female	1.28 ± 0.08	1.52 ± 0.11^a^	1.18 ± 0.13^b^	1.37 ± 0.16
Male	*0.72 ± 0.05*	*0.73 ± 0.06*	*0.79 ± 0.1*	*0.59 ± 0.07*
Testosterone (ng/ml)				
Female	0.11 ± 0.01	0.11 ± 0.01	0.12 ± 0.01	0.12 ± 0.01
Male	*0.29 ± 0.05*	*0.34 ± 0.03*	*0.34 ± 0.06*	*0.36 ± 0.05*
Oestradiol (pg/ml)				
Female	5.7 ± 2.4^a^	1.5 ± 0.9^b^	7.6 ± 4.4^a^	4.0 ± 1.5
Male	*19.1 ± 3.7* ^*a*^	*33.9 ± 4.6* ^*b*^	*12.2 ± 1.9* ^*a*^	*16.8 ± 3.2* ^*a*^
Inhibin A (pg/ml)				
Female	7.69 ± 0.69^a^	12.99 ± 1.89^b^	8.69 ± 1.69^a^	9.95 ± 1.60
Male	*173 ± 6*	*168 ± 7*	*152 ± 10*	*159 ± 6*

FSH, follicle‐stimulating hormone; LH, luteinising hormone.

CC, control; CT, raised on control pastures, then transferred and maintained on biosolids‐treated pastures; TC, biosolids‐treated, then transferred and maintained on control pastures; TT, continuous biosolids treated.

Different superscripts denote differences at P < 0.05 across exposure groups. Italics represent significant differences at P < 0.05 between sexes within each exposure group.

### Timing of maternal exposure and effects on foetal HPG axis

#### GnRH and GnRH receptor (GnRHR) expression

**Figure 1:**
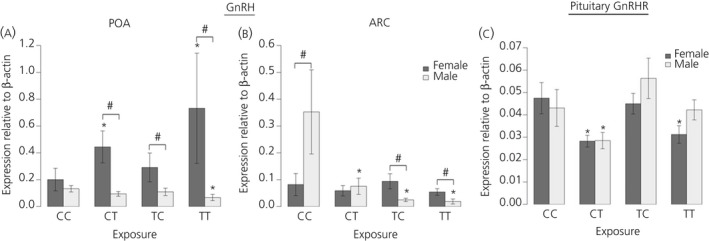
Maternal exposure to biosolids chemicals before conception only (TC), during pregnancy only (CT) or life‐long (TT) has sex‐ and region‐ specific effects on foetal male and female gonadotrophin‐releasing hormone (GnRH) mRNA expression in the hypothalamus. In the preoptic area (POA) (a), a divergent effect on expression can be seen between males (suppression) and females (increase) in the TT group. Similarly in the arcuate nucleus (ARC) (b), exposure is associated with suppression in males but no effect in females. In the pituitary gland (c), the effect of exposure on GnRH receptor (GnRHR) expression is also sexually dimorphic, with a greater effect in females compared to males relative to unexposed foetuses (CC); n = 12 (CC and TT); n = 11 (CT); n = 10 (TC). *Significant difference compared to respective control group (P < 0.05). #Significant sex difference within a particular group (P < 0.05).

Hypothalamic GnRH mRNA expression levels in control (CC) males were not statistically different from females in the POA, although, in the ARC, *GnRH* mRNA expression was significantly (P < 0.05) higher in males compared to females (Fig. 1 a). Biosolids exposure had significant effects on GnRH mRNA expression in both the POA and ARC, with the effects differing between the two nuclei and not following the same trend in males and females.

In the hypothalamus in female foetuses, the effects of exposure to biosolid EDs were observed only in the POA. The pregnancy exposure alone group (CT) and life‐long exposure (TT) groups had significantly (P < 0.05) higher expression of *GnRH* mRNA compared to the CC group (Fig. 1 a). In the ARC, there was no effect of treatment on *GnRH* expression in female foetuses.

In males, effects were observed in both hypothalamic regions. *GnRH* mRNA expression in the POA was significantly (P < 0.05) reduced in the TT relative to the CC group In the ARC, *GnRH* mRNA expression in male foetuses was significantly (P < 0.05) lower in all biosolid exposed (CT, TC, TT) groups relative to the CC group. In the ARC, in which males from the CC group had significantly (P < 0.05) higher *GnRH* expression than females, this sex difference was reversed in the preconception only exposure (TC) and TT groups, such that *GnRH* mRNA expression was significantly (P < 0.05) lower in males compared to females (Fig. 1 b).

In the pituitary gland, there were no significant sex differences in *GnRHR* expression in any of the treatment groups (Fig. 1 c). In female foetuses, *GnRHR* mRNA expression was significantly (P < 0.05) lower in the CT and TT groups relative to the CC group (Fig. 1 c). In male foetuses, the only significant difference in *GnRHR* mRNA expression was seen in the CT group, in which expression was significantly (P < 0.05) lower than the CC group, as found in females (Fig. 1 c).

#### KISS1 and KISSR

**Figure 2:**
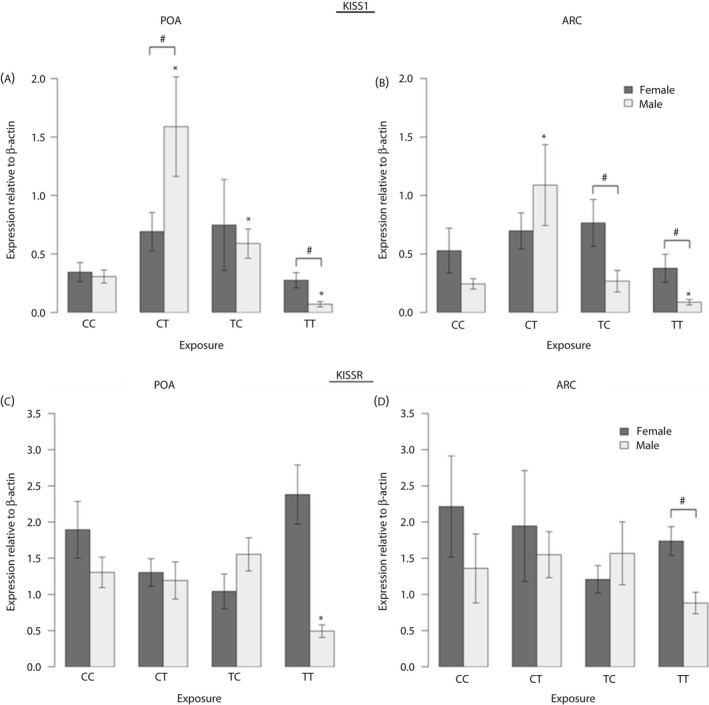
Maternal exposure to biosolids chemicals before conception only (TC), during pregnancy only (CT) or life‐long (TT) has sex‐ and region‐ specific effects on foetal male and female KiSS1 and KiSS1R mRNA expression. For KiSS1 (a, b) and KiSSR expression (c, d), a greater number of effects are seen in males compared to females. Interestingly, for KiSS1 expression in both the preoptic area (POA) (a) and arcuate nucleus (ARC) (b) regions of hypothalamus, life‐long exposure reduced expression whereas pregnancy only exposure (CT) caused an increase in expression relative to unexposed foetuses (CC). For both KISS1 and KISSR, life‐long exposure resulted in sexually dimorphic expression levels that were not seen when comparing control males and females; n = 12 (CC and TT); n = 11 (CT); n = 10 (TC). *Significant difference compared to respective control group (P < 0.05). #Significant sex difference within a particular group (P < 0.05).

There was no sex difference in hypothalamic *KISS1* mRNA expression in the POA or ARC (Fig. 2 a,b) within the control group.

In the POA of females, there were no significant effects of biosolids exposure on *KISS1* mRNA expression (Fig. 2 a). By contrast, in the POA of male foetuses, *KISS1* mRNA expression was significantly (P < 0.05) increased in the TC and CT compared to the CC group (Fig. 2 a) but was significantly (P < 0.05) reduced in the TT group compared to the CC group (Fig. 2 a).

In the ARC, there was no effect of biosolids exposure on *KISS1* expression in female foetuses; however, in the males, *KISS1* mRNA expression was significantly (P < 0.05) higher in the CT group, and significantly (P < 0.05) lower in the TT group, relative to the CC group (Fig. 2 b).

For hypothalamic *KISS1R* mRNA expression, there were no sex differences in mRNA expression in the CC groups in either the POA or the ARC (Fig. 2 c,d). In the POA in female foetuses, there was no significant effect of biosolids exposure on *KISSR* mRNA expression levels. In male foetuses, however, *KISSR* mRNA expression in the POA was significantly (P < 0.05) lower in the TT group relative to CC group (Fig. 2 c). In the ARC, there were no statistically significant effects on *KISSR* expression in either males or females. In both the POA and the ARC in the TT group, females expressed significantly higher levels of *KISSR* compared to males (Fig. 2 a).

#### Gonadotrophins

As reported previously and summarised in Table 1, there was no effect of treatment on plasma LH concentrations in the female foetuses. In males, however, LH was significantly higher in the CT and TC groups compared to the CC group. When comparing LH concentrations between males and females, there was no significant difference in the CC and TT groups but both CT and TC males had significantly higher LH concentrations compared to females of the same treatment groups. In the females, the FSH concentration in the TT group was significantly higher than the CT group, which had the lowest FSH concentration of all groups but was not different to the CC or TC groups. In male foetuses, FSH concentrations were significantly lower than the females in all of the treatment groups but FSH concentrations did not differ between treatment groups.

#### Gonadal hormones

As expected, in all groups, male foetuses had significantly (P < 0.05) higher testosterone concentrations compared to females. However, the plasma testosterone concentrations in both female and male foetuses were not significantly different between treatment groups. In female foetuses, the oestradiol concentration was significantly (P < 0.05) higher in the CT group than in the CC and TT groups. Plasma inhibin A concentrations in females were significantly (P < 0.05) higher in the TT group than in both CC and CT groups but not the TC group. In male foetuses, inhibin A concentrations were not significantly affected by treatment but were significantly (P < 0.05) higher than the females in all exposure groups.

#### Timing of maternal exposure and effects on foetal oestrogen and aryl hydrocarbon receptor mRNA expression

**Figure 3:**
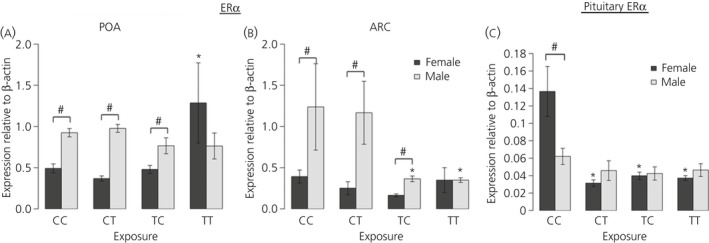
Maternal exposure to biosolids chemicals before conception only (TC), during pregnancy only (CT) or life‐long (TT) has sex‐ and region‐ specific effects on foetal male and female oestrogen receptor (ER)α mRNA expression in the (a) preoptic area (POA) and (b) arcuate nucleus (ARC) regions of hypothalamus and (c) in the pituitary gland relative to each other and to unexposed foetuses (CC). In both the POA and ARC, there is sexually dimorphic expression between CC males and females that is subsequently lost in the TT group. Interestingly, pituitary gland ERα expression is only affected in females exposed to biosolids chemicals; n = 12 (CC and TT); n = 11 (CT); n = 10 (TC). *Significant difference compared to respective control group (P < 0.05). #Significant sex difference within a particular group (P < 0.05).

In both the POA and ARC regions of the hypothalamus, control males had significantly (P < 0.05) higher *ER*α mRNA expression compared to control females (Fig. 3 a,b). Effects of exposure to biosolids on foetal *ER*α mRNA expression were again different between the POA and ARC regions and did not follow the same trend in males and females.

In the POA in males, there was no significant effect of exposure to biosolids on *ER*α mRNA expression (Fig. 3 a), whereas in female foetuses, the TT group had significantly (P < 0.05) higher expression of *ER*α mRNA in the POA relative to the CC group (Fig. 3 a). In the TT group, there was also no significant sex difference in *ER*α mRNA expression, which contrasts with the CC, CT and TC groups in which significantly (P < 0.05) higher *ER*α mRNA expression was seen in males compared to females for both POA and ARC.

In the ARC, biosolids exposure did not affect *ER*α mRNA expression in females but, in the males, *ER*α mRNA expression was significantly (P < 0.05) lower in the TC and TT groups relative to the CC group (Fig. 3 b). In the TT group, as in the POA, this meant that the significantly (P < 0.05) higher *ER*α mRNA expression seen in the ARC in CC males compared to females was no longer evident (Fig. 3 b).

In the pituitary gland, a significant sex difference was seen in *ER*α mRNA expression in the CC group, with levels being significantly lower (P < 0.05) in males compared to females (Fig. 3 c); however, this difference was not evident in any of the biosolids exposed groups, and *ER*α mRNA expression was significantly (P < 0.05) lower in female foetuses from the CT, TC and TT groups compared to the CC group (Fig. 3 c). There was no significant difference in *ER*α mRNA expression in the ARC between any of the male exposure groups.

**Figure 4:**
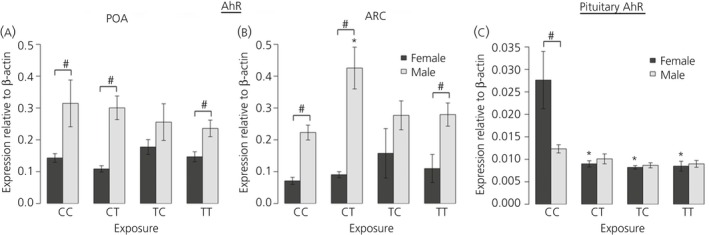
Aryl hydrocarbon receptor (AhR) mRNA expression in the (a) preoptic area (POA) and (b) arcuate nucleus (ARC) regions of hypothalamus. An effect of exposure was only seen in pregnancy only (CT) exposed males in the ARC relative to controls (CC) with no effect of exposure in the preconception (TC) or life‐long (TT) exposure groups. Interestingly, female foetal hypothalamic AhR expression was not affected by treatement, which contrasts with the effects on AhR expression in the pituitary gland (c) where expression was reduced in all exposed groups relative to controls, whereas male AhR expression was unaffected; n = 12 (CC and TT); n = 11 (CT); n = 10 (TC). *Significant difference compared to respective control group (P < 0.05). #Significant sex difference within a particular group (P < 0.05).

For *AhR*, in both hypothalamic regions, CC males had significantly (P < 0.05) higher aryl hydrocarbon receptor (AhR) expression compared to females (Fig. 4 a,b).

In the POA, there was no effect of biosolids exposure on *AhR* mRNA expression in either sex (Fig. 4 a). In the ARC, in the females, there was a similar absence of effect of biosolids exposure on AhR mRNA expression, whereas, in males, *AhR* mRNA expression was significantly (P < 0.05) increased in the CT relative to the CC group, although this was not evident in the TC and TT groups, relative to the controls (Fig. 4 b). There was a significant sex difference in pituitary gland *AhR* expression in the CC group, with expression being notably higher in female foetuses (P < 0.05). However, this difference was not evident in any of the biosolids exposed groups (Fig. 4 c). In female foetuses, *AhR* mRNA expression was significantly lower in the TC, CT and TT groups compared to the CC group. There was no effect of biosolids exposure on *AhR* mRNA expression in male foetal pituitary glands (Fig. 4 c).

## Discussion

This is the first study to address the effects of the timing of maternal exposure to environmentally relevant chemical mixtures on the foetal reproductive neuroendocrine system. The findings from this ovine model are significant for the following reasons. They show that: (i) the effects of maternal exposure to mixtures of ECs in biosolids on the foetal neuroendocrine system are sexually dimorphic; (ii) the timing and duration of maternal EC exposure is critical in determining its effects on the foetal neuroendocrine system; in particular EC exposure prior to conception, but then not during pregnancy, can affect development of the foetal reproductive neuroendocrine system; (iii) the effect of maternal exposure to EC mixtures during pregnancy alone on the foetal neuroendocrine system can be different compared to those seen when maternal exposure has been life‐long (i.e. prior to and during pregnancy); and (iv) life‐long maternal exposure to chemical mixtures does not necessarily mean greater/more effects on gene expression in the foetal neuroendocrine system.

Specifically, the inhibitory effects of EC exposure on *GnRH* and *KISS1* (POA and ARC), *ER*α (ARC) and *KISSR* (POA) mRNA expression in the male hypothalamus are more pronounced and, indeed, were in the opposite direction relative to the observed increase in *GnRH* and *ER*α mRNA expression within the female, and were not suppressed but were increased in the POA. The suppression of *KISS1* expression in foetal males is consistent with our previous studies and studies in rodents exposed to endocrine disruptors during foetal development 47, 48 and demonstrates that the kisspeptin system is a potential target for endocrine disruption. Although our results in females are in contrast to those in males, they are consistent with previous studies in rodents exposed prenatally. The most striking similarity was an increase in *GnRH* mRNA expression that has been observed in the POA in response to *in utero* exposure to chemical mixtures (PCBs) in Aroclor 1221, methoxyclor and chloropyrifos 45, and increased *ER*α mRNA expression was observed following exposure to bisphenol A in female rodents 49 and sheep 50. However, these findings contrast with those of Mahoney and Padmanabhan 50, who showed a consistent suppression of *GnRH* expression in the POA following prenatal exposure to BPA and methoxyclor in both male and female sheep. Because biosolids represent largely unquantifiable low‐level mixed exposure, it is difficult to draw direct comparisons to the effects on the foetal hypothalamus of other ‘known dose’ exposure studies on exposures in different species. Furthermore, there is a cocktail effect between the different molecules from a mixture that does not boil down to the sum of the effects but may be responsible for divergent/inverse effects of single molecules. Nevertheless, the observation that GnRH expression is altered by life‐long low‐level maternal EC exposure in the current study is important because increased GnRH expression may be a contributory factor in precocious puberty in females 45, 46 and therefore may have implications for later adult reproductive function.

For some of the hypothalamic genes investigated in the present study (*GnRH* (POA), *KISS1* and *KISSR*), mRNA expression showed sex‐specific disturbances after life‐long EC exposure. However, for others, the sexually differentiated pattern seen in controls was reversed (*GnRH*, ARC) or lost (*ER*α) in the foetuses of ewes that had been exposed to ECs throughout their lifetime. These effects on sexually differentiated gene expression were not seen in our previous study that used the same life‐long EC exposure paradigm. Although this could be a result of lower numbers of animals in our previous study, we cannot rule out the possibility that animals in the two studies were exposed to different chemical concentrations or chemical mixtures as a result of variation in biosolids composition or grazing patterns.

The sexually dimorphic effects of EC exposure were not limited to the hypothalamus, because life‐long EC exposure also significantly affected mRNA expression in the pituitary gland. However, in this tissue, the effects were more pronounced in females, in which expression of all investigated genes (*GnRHR*,* ER*α, *AhR*) was significantly reduced compared to males in which GnRHR mRNA expression was reduced only in the pregnancy exposed group (CT). The results for GnRHR and ERα in the female pituitary gland are consistent with our previous studies in foetuses from life‐long biosolids exposed mothers 32, 33.

These results provide compelling evidence that the foetal neuroendocrine system is altered following life‐long maternal exposure to EC mixtures in biosolids and, critically, that the reproductive neuroendocrine axis of male and female foetuses may react differently to EC exposure. Sexually dimorphic differences in the brain are present in foetal life, and are found in almost every region of the brain, particularly the hypothalamus 51, 52. It is therefore not surprising that the effects of life‐long chemical exposure on expression levels of key reproductive neuroendocrine systems are sexually dimorphic. Numerous studies in rodents have demonstrated sex‐specific endocrine disrupting effects on brain development, as well as differential effects on different brain regions, as also shown in the present study 53. In particular, studies on rodents exposed, during gestation, to the PCB mixture Aroclor 1221 showed altered expression of numerous oestrogen‐sensitive genes in the anteroventral periventricular nucleus in females but not in males 47; in contrast, the ARC was affected in males but not females. Although there are critical species differences to be taken into account, it is well established that sexual differentiation of the hypothalamus is an oestrogen‐dependent process 41. During the developmentally critical sensitive period, oestrogen induces a permanent alteration in the neural control of physiological functions that persist into adulthood 54, 55. Accumulating evidence suggests an important role for oestrogen and kisspeptin receptors in the hypothalamus for sexual differentiation of the brain and behaviour 56. Many of the chemicals found in biosolids have been shown to have oestrogenic effects and the present study provides evidence that oestrogen sensitive genes in the hypothalamus and pituitary gland may be a target for EDCs, which could potentially have long‐term effects on neuroendocrine function.

The present study specifically addressed the question as to whether the timing of maternal exposure is critical for determining subsequent effects of EC exposure on the foetal neuroendocrine axis. In the groups in which maternal exposure took place during pregnancy alone, significant sexually differentiated, region‐specific effects on the expression of key neuroendocrine genes were also observed. However, the total number of changes in mRNA expression (relative to controls) was less in the pregnancy alone exposure group compared to the continuous maternal exposure group, as might be expected given that exposure was shorter. The differential effects between the pregnancy exposure alone and life‐long exposure groups could be related to adaptive responses such as increases in xenobiotic metabolising enzyme expression or activity 57, which may have been induced by the preconception exposure and then maintained during pregnancy, thereby mitigating the effects of biosolids exposure on the foetus.

When maternal EC exposure occured only prior to pregnancy, this also resulted in lower expression of mRNA for *GnRH* and *ER*α in the ARC (males only). Similarly, for the pituitary gland, preconception biosolids exposure significantly reduced *ER*α and *AhR* expression to an extent the same as that resulting from life‐long exposure in females. However, the exposure effects on other genes were different; for example, POA *KISS1* mRNA expression in males was increased after preconception exposure, whereas life‐long exposure reduced *KISS1* mRNA expression. These results are consistent with our previous findings of effects in the ovaries of foetuses from mothers exposed to biosolids only prior to conception 34. This result is of specific concern because foetal EC exposure in this paradigm is likely to be mostly attributable to mobilisation of chemicals from maternal fat stores. Worryingly, these would have been laid down prior to mating and then released later in pregnancy when energy demands in the mother are increased. As might be expected, the overall number of effects of exposure was lower in the foetuses born to mothers exposed to ECs prior to pregnancy, and the pattern of changes observed, differed from that seen in the life‐long maternal exposure group. This is likely to reflect the fact that the foetus may be exposed to varying levels and mixtures of ECs as they are released from adipose stores throughout several important windows of development. This is important because it has recently been shown that there is differential temporal sensitivity across gestation where maternal exposure to biosolids during different gestational windows has varying effects on the foetal ovary 24, with this being difficult to predict or measure.

Another explanation is that the oocyte/embryo product during the pre‐and peri‐conceptional period was affected by EDC exposure. Profound epigenetic modifications to the genome occur in the late folliculogenesis and in early embryo as a normal part of development. Recent evidence suggests that environmental signals acting during early development may also result in epigenetic changes, which may play a role in mediating the association between early‐life exposures and later phenotype 58, 59. Most evidence of periconceptional ‘programming’ has emerged not only from maternal nutritional models, but also other *in vivo* and *in vitro* conditions, including assisted reproductive treatments, showing consistent outcomes 60. To support the hypothesis of a release of stored EDCs by the mother or epigenetic alteration of the oocyte/zygote, crossed embryo transfer would be necessary.

The mechanisms by which chemicals in biosolids may affect neuroendocrine function are likely to be numerous given the mixture of different chemicals present in biosolids and the importance of the timing of exposure. Determination of the potential mechanisms underlying neuroendocrine disruption is therefore difficult, although, given the similar effects of exposure on both ERα and AhR, the present study would suggest that the activation of both of these receptors may be involved. Several chemicals found in biosolids, such as PCB congeners, are well known ligands for AhR, which is involved in the detoxification of endogenous and exogenous substrates by cytochrome P450 enzymes and cell regulation, oxidative stress and apoptosis 61. Several previous studies have demonstrated that AhR is expressed in the hypothalamus and pituitary gland and activated by endocrine disruptors in these tissues 62, 63, 64. In addition, AhR expression in the hypothalamus is sexually dimorphic during late gestation in rodents 64, with males expressing higher levels of AhR compared to females, which is in agreement with the results of the present study. Moreover, in the present study, pregnancy exposure to biosolids also increased AhR expression in males but not females, which is also in accordance with previous studies of mixed prenatal PCB exposure in rats 64. The similar effects of biosolids exposure on AhR and ERα are perhaps not surprising because activation of the AhR pathway can interfere with ERα pathways through a number of mechanisms. There are complex interactions between AhR, ERα and gonadotrophin release and synthesis by direct actions on gonadotrophs 63. Induction of ERα and AhR may represent putative pathways through which biosolids exposure may alter neuroendocrine hormone release.

A critical question is whether alterations to the expression of genes involved in regulation of gonadotrophin release are adaptive or may impact on normal reproductive function in later life. In the present study, gonadotrophin concentrations were quantified in the EC exposed foetuses and, although it is recognised that foetal gonadotrophin secretion may not mirror what would be seen in the adult, it was interesting to note that gonadotrophin concentrations were significantly affected by EC exposure; however, the present study measured plasma concentrations at a single time point, and it is unclear how this concentration corresponds to the pulsatility of LH secretion, which is likely to be different in each animal. In addition, we have previously shown that, in adult males exposed to biosolids *in utero*, significantly altered testis structure is not reflected in gonadrotrophin or steroid hormone concentrations, which were unaffected. Although the present study cannot confirm whether altered neuroendocrine gene expression in response to biosolids exposure is related to changes in gonadotrophin release, our previous study would suggest that altered foetal reproductive neuroendocrine development may have long‐term consequences for reproductive function, at least in males; however, this warrants further investigation.

The results of the present study not only support and extend our previous neuroendocrine findings 32 but, crucially, also demonstrate that foetal sex and the timing of maternal exposure are critical when assessing the effects of exposure to low levels of mixtures of chemicals.

## Supporting information


**Fig. S1.** Diagrammatic summary of the study design.

**Table S1.** Effects of chemical cocktails in sewage sludge on morphological and endocrine characteristics maternal ewes on day 110 of pregnancy.
