# Supplementary material for: Timing of Maternal Exposure and Foetal Sex Determine the Effects of Low‐level Chemical Mixture Exposure on the Foetal Neuroendocrine System in Sheep
Source: J Neuroendocrinol. 2016 Dec 14;28(12):10.1111/jne.12444. doi: 10.1111/jne.12444 (PMC5621486; doi:10.1111/jne.12444)
Supplement: Supplementary file 1 — Fig. S1. Diagrammatic summary of the study design. [file JNE-28-0-s001.docx]

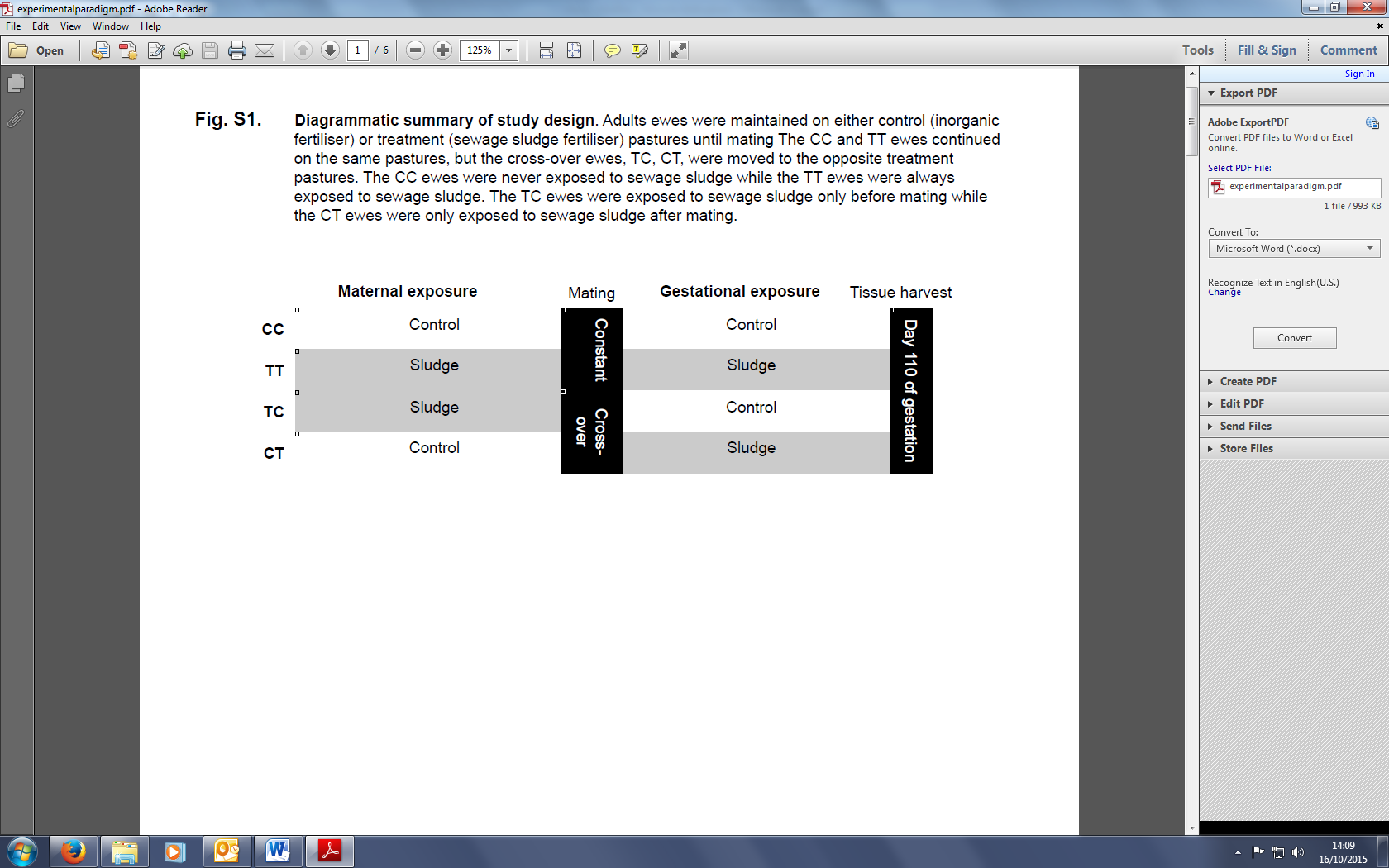
**Fig S1.** Diagrammatic summary of study design. Four experimental exposure groups were set up in parallel. 2 groups of ewes were maintained on either control (inorganic, CC) and 2 groups maintained on o biosolids (sewage sludge, TT) treated pastures until mating. One group of CC and and one group of TT ewes were continued on the same pastures, but the other groups of ewes, (TC, CT) were crossed over to the opposite treatment pastures at the time of conception. The CC ewes were never exposed to biosolids treated pastures while the TT ewes were always exposed to biosolids pastures. The TC ewes were exposed to biosolids only before mating while the CT ewes were only exposed to biosolids after mating.
