# Supplementary material for: Timing of Maternal Exposure and Foetal Sex Determine the Effects of Low‐level Chemical Mixture Exposure on the Foetal Neuroendocrine System in Sheep
Source: J Neuroendocrinol. 2016 Dec 14;28(12):10.1111/jne.12444. doi: 10.1111/jne.12444 (PMC5621486; doi:10.1111/jne.12444)
Supplement: Supplementary file 2 — Table S1. Effects of chemical cocktails in sewage sludge on morphological and endocrine characteristics maternal ewes on day 110 of pregnancy. [file JNE-28-0-s002.docx]

**Supplementary Table S2**

Effects of chemical cocktails in sewage sludge on morphological and endocrine characteristics maternal ewes on day 110 of pregnancy. Values are mean±SEM. Different superscripts denote differences at p<0.05.

| Treatment groups | Constant exposure profile | |  | Cross-over exposure profile | |
| --- | --- | --- | --- | --- | --- |
|  | CC (n=12) | TT (n=12) |  | CT (n=11) | TC (n=10) |
| Morphology |  |  |  |  |  |
| Live weight (kg) | 82±3^a^ | 88±2^a^ |  | 82±3^ab^ | 90±1^c^ |
| Body condition score | 2.3±0.1^a^ | 2.6±0.1^b^ |  | 2.4±0.1^ac^ | 2.5±0.1^bc^ |
| Number of fetuses | 2.1±0.2^a^ | 2.2±0.2^a^ |  | 2.0±0.2^a^ | 2.5±0.2^a^ |
| % of male fetuses | 40±10^a^ | 56±9^a^ |  | 39±13^a^ | 51±11^a^ |
| Endocrinology |  |  |  |  |  |
| Estradiol (pg/ml) | 2.5±0.7^a^ | 0.7±0.1^b^ |  | 3.2±1.1^ac^ | 2.8±1.0^a^ |
| Inhibin A (pg/ml) | 46±24^a^ | 46±34^a^ |  | 62±40^a^ | 36±14^a^ |
| FSH (ng/ml) | 0.52±0.07^a^ | 0.37±0.04^a^ |  | 0.40±0.05^a^ | 0.40±0.02^a^ |
| LH (ng/ml) | 1.15±0.15^a^ | 1.18±0.10^a^ |  | 0.95±0.07^ab^ | 0.83±0.07^b^ |
| Testosterone (ng/ml) | 0.09±0.01^a^ | 0.11±0.01^b^ |  | 0.09±0.01^a^ | 0.09±0.01^a^ |
| Progesterone (nmol/l) | 96±8^a^ | 156±15^b^ |  | 118±13^ac^ | 129±8^bc^ |
